# RNF7 Facilitated the Tumorigenesis of Pancreatic Cancer by Activating PI3K/Akt Signaling Pathway

**DOI:** 10.1155/2023/1728463

**Published:** 2023-01-04

**Authors:** Hao Hua, Huahua Xie, Jian Zheng, Linhan Lei, Zilei Deng, Chao Yu

**Affiliations:** ^1^Department of Hepatic-Biliary-Pancreatic Surgery, The Affiliate Hospital of Guizhou Medical University, Guiyang, Guizhou, China; ^2^School of Clinical Medicine, Guizhou Medical University, Guiyang, Guizhou, China

## Abstract

RING finger protein-7 (RNF7) functions as a positive regulator in the progression of multiple malignancies. However, the underlying mechanism by which RNF7 contributes to pancreatic cancer (PC) is lacking. Quantitative real-time polymerase chain reaction (qRT-PCR) was used to test the level of RNF7expression in PC cell lines and tissues. The role of RNF7 in PC tumorigenesis was analyzed by Cell Counting Kit-8 (CCK-8). 5-Ethynyl-20-deoxyuridine (EdU), wound-healing/Transwell assays, as well as a subcutaneous tumorigenesis model were constructed to assess the role of RNF7 in PC cells. The association between RNF7 and PI3K/Akt signaling were assessed by western blot and further confirmed by rescue experiments. The PC patients with upregulated expression of RNF7 had poor survival. Overexpression of RNF7 significantly facilitated PC proliferative and migrative and invasive properties in vitro and vivo; however, knockdown of RNF7exhibited the opposite results. Mechanistically, RNF7 promoted PANC-1 and SW1990 cell growth through impacting the activation of the PI3K/Akt signaling pathway. Our data demonstrated that RNF7 promoted PC tumorigenesis via activating the PI3K/Akt signaling pathway and might be regarded as one of the potential therapies to PC.

## 1. Introduction

Pancreatic cancer, one of the most aggressive and intractable cancers, possesses an extremely low 5-year survival rate [1, 2]. Most patients having few manifestations are challenging to diagnose at a nearly stage of the disease, and the most common signs are jaundice (55%) and hepatosplenomegaly (39%) [3]. Consequently, the disease develops rapidly. The major curative treatment for pancreatic cancer is surgery, and inoperable PC is refractory to radiotherapy and chemotherapy, which have limited efficacy [4]. Although significant advances in therapy have been acquired, the prognostic effect remains dissatisfied. In addition, it is difficult to deal with PC because of its local destructive capacity and propensity for invading the surrounding organs and blood vessels. High-recurrence rate and metastasis rate are the main reasons for treatment failure of PC [5]. Herein, better understanding of pancreatic cancer pathogenesis is beneficial for PC treatment.

RING finger protein-7 (RNF7), also known as SAG (sensitive to apoptosis gene), ROC2 (regulator of cullins 2), or Rbx2 (RING-box 2), is a member of evolutionarily conserved gene family with a molecular weight of 12.6 kDa [6]. RNF7 is generally overexpressed in human skeletal muscles, heart, testis, and other many human cancers [7]. As a composition of E3 ubiquitin ligases, RNF7 also possesses a E3 ubiquitin ligase activity and may intermingle with massive cellular proteins [8]. Early study showed that RNF7 was emerged as a redox-inducible antioxidant protein, which could inhibit cells from apoptosis caused by oxidation [9]. It was found that RNF7 act an imperative role in tumor proliferation and radiation-resistance [7] through inactivating NF-*κ*B and mTOR pathway [10] or assembling tumor suppressive proteins, including NF1, DEPTOR, procaspase-3, p21, p27, NOXA, and BIM [11]. Beyond that, RNF7 transgenic expression is not only considered to be implicated within skin tumorigenesis induced by DMBA-TPA [12] and UVB-radiation [13] but also the lack of RNF7 in mouse embryonic fibroblasts harbors an association with the inhibition of Kras^G12D^-induced immortalization and transformation [14]. Furthermore, in the early stages of tumor formation, RNF7 can negatively influence differentiation, proliferation, and cell growth through targeting c-Jun/AP-1, and in the later stage, the opposite result occurs by targeting I*κ*B*α*/NF-*κ*B based on the availability of F-box proteins [12]. Moreover, Xiao et al. elucidated that dysregulated expression in RNF7 affects proliferation and tumorigenesis, with this trend occurring within prostate cancer by inactivating ERK 1/2 pathway, suggesting that RNF7 can be used as tumor markers for PC treatment [15]. Nevertheless, the effect of RNF7 in a variety of tumors, including PC, has not been extensively studied. Especially, the potential mechanisms employed by RNF7 to be involved in PC malignancy are still not clear.

In our research, we explored the effects and mechanism of RNF7 in PC. The results verified that RNF7 promoted proliferation, migration, and invasion of PC cells. Besides, we unraveled that knockdown of RNF7 suppressed tumorigenesis and progression in PC. The purpose of our findings is to supply us with a novel insight into understanding the pathogenesis of PC, so as to supply us with an available role for the diagnosis and therapy of PC.

## 2. Methods

### 2.1. Human Specimens

Forty-seven PC samples in the Affiliated Hospital of Guizhou Medical University were collected between 2017 and 2022. The study was performed after approval by the Institutional Ethical Board of our affiliated hospital. All enrolled individuals provided informed consent. Paired distant normal tissues away from the tumor margins at least 5 cm were served as controls.

### 2.2. Cell Lines and Transfection

AsPC-1, BxPC-3, Capan-1, Capan-2, MIA PaCa-2, PANC-1, SW1990, and human normal pancreatic ductal epithelial cell line (HPDE) were obtained from American Type Culture Collection (Manassas, VA, USA). PANC-1 and SW1990 cells were cultured in Dulbecco's Modified Eagle Medium (DMEM) supplemented with 10% heat-inactivated fetal bovine serum (FBS; Gibco, CA, USA). All cells were grown at a humidified temperature (37°C) and in a moist incubator stabilized at 5% CO_2_.To knock down endogenous RNF7, PC cells were selected for subsequent transfection using Lipofectamine 3000 transfection reagent (Invitrogen) to improve the transfection efficiency.

### 2.3. RNA Extraction and Quantitative Real-Time PCR

TRIzol reagent (Catalog No. 15596018, Invitrogen, USA) was used for the isolation of total RNA from PANC-1 and SW1990 cells or PC tissues based on its protocol. Subsequently, we used a First-Strand cDNA Synthesis Kit (Catalog No. K1622, Invitrogen, USA) to obtain the complementary DNA (cDNA). For real-time PCR (qRT-PCR), amplifications were performed by the real-time PCR Master Mix (SYBR Green; Vazyme Biotech Co., Ltd). We used 2 − ΔΔCt method to analyze the mRNA expression. *β*-Actin expression was used as internal controls. The primer sequence of the genes was shown:

*β*-Actin, forward primer:CATGTACGTTGCTATCCAGGC

Reverse primer:CTCCTTAATGTCACGCACGAT

RNF7, forward primer:ACCCTGCGTCCTTTCTTCG

Reverse primer:GGCACAGGTATCGCACTCAA

### 2.4. Western Blotting

All tissue/cell line samples were applied to extract protein by using RIPA buffer (KenGEN, China), and the protein was quantified by using bicinchoninic acid (BCA, Boster, China). The remaining specific steps of western blot (WB) refer to reported previously [16].

### 2.5. Cell Counting Kit-8 (CCK-8) Assay

The transfected cells were cultured into 96-well plates with 3000 cells per well and cultured at 37°C with 5% CO_2_. Consequently,10 *μ*L of CCK-8 reaction reagent were added at a specific time (0, 24, 48, 72, and 96 h). And then, after 2 hours incubation, absorbance (OD) values at 450 nm were determined by enzyme reader. Data were recorded, and cell proliferation was detected.

### 2.6. 5-Ethynyl-20-Deoxyuridine (EdU) Assay

Cells from each group of PANC-1 and SW1990, seeded into 6-well plates (1.5 × ­10^4^ cells/well), were cultured with 200 *u*L of EdU medium (5 *u*M) for 2 h. Subsequently, cells were fixed in 4% paraformaldehyde for 30 min at room temperature. After PBS wash-step, Hoechst 33,342 was added for proliferating cells stained with Apollo Dye Solution for 30 minutes. Finally, images of cells were photographed, and cells were counted under laser confocal microscopy.

### 2.7. Wound-Healing Assay

After the transfected PC cells were planted in 6-well plates, the confluent cell monolayer was created via using a 200 *u*L pipette tip once grew to 80% confluences. The floating cells were washed away with 1×PBS. A phase contrast microscope and ImageJ (Fiji) software were used to monitor the area of cell migration into the wound and capture the healed wound width at different times. Linear changes were monitored from the initial wound width at 0 h, and the cellular migrative ability was observed.

### 2.8. Transwell migration Assay and Matrigel Invasion Assay

Assays for cell migration and invasion were carried out in 8 *μ*M Transwell chambers (Catalog No. 3422, Corning, USA). For the migration experiment, the cells were put into the top chamber, and 0.5 mL of medium with 10% FBS was poured into the lower chamber, and for 24 h, cells were incubated at 37°C in 5% CO_2_. The upper side of the membrane at the bottom of the Transwell chamber was coated with Matrigel for the invasion experiment and was incubated at 37°C for 60 minutes. Next, an approximate 200 *u*L suspension containing 1 × 10^4^ SW1990 or PANC-1 cells was suspended into the top chamber, and 0.5 mL medium with 10% FBS was introduced to the bottom chamber. Cells adhering to the uppermost surface of the membrane were washed thoroughly and discarded after 24 h of incubation, whereas invasive or migrated cells sticking to the bottom surface were colored with 2% crystal violet and fixed with 4% paraformaldehyde.

Transwell assay pictures were acquired by means of a microscope (100x magnification) to record the stained lower chambers.

### 2.9. Immunohistochemistry

Human and mouse tissue was fixed and sliced into 5 *μ*m-thick slices before rehydration, deparaffinized, and cleaned. Antigen retrieval was conducted using a citrate antigen retrieval solution, and endogenous peroxidase activity was suppressed. Then, sample sections were immunostained with primary antibodies (Ki67 and PCNA) and incubated with horseradish peroxidase-conjugated secondary antibody overnight at 4°C. Furthermore, hematoxylin was used to visualize and counterstain the slices. In the end, samples were dehydrated, rendered transparent, and sealed for taking pictures. The degree of immunostaining of indicated proteins was assessed based on the percentages of tumor cells that stained positively and the strength of the staining.

### 2.10. Development of the PC Animal Mouse Model

To develop the PC mouse subcutaneous model, female thymic nude mice ages 5 to 6 weeks was randomly categorized into two groups (*n* = 4) and were seeded with cells with transfection of RNF7 overexpression (Lv-RNF7) or control (Lv-control) lentivirus purchased from GeneChem (Shanghai, China). And then, the cells were inserted into the right armpit of mice. 2 months later, visible tumors of mice killed by cervical dislocation were embedded into paraffin for immunohistochemical (IHC) staining and weighed. Tumors were assessed along two orthogonal axes (a: length; b: width), and the lump volume and weight of the mice were recorded weekly. All the animal experiments were carried outconsistently with the institutional guidelines and approved by the Animal Committees of Affiliated Hospital of Guizhou Medical University.

### 2.11. Bioinformatics Examination

We analyzed the relative genes of RNF7 using the Gene Expression Profiling Interactive Analysis (GEPIA) database. Meanwhile, the underlying biological function of RNF7 in PC was investigated via Gene Ontology (GO) and enrichment assessment using the Kyoto Encyclopedia of Genes and Genomes (KEGG) .To check, the clusterProfiler R package was used to utilized. Padj <0.05 was considered a significant difference, and the R package “ggplot2” was applied to display the statistical enrichment of genes with differential expression in KEGG pathways and GO domains.

### 2.12. Statistical Study

The measurement data of each experimental result was expressed as the mean ± S.D. A statistical comparison study between two groups was performed with one-way ANOVA, and an independent sample Student's *t*-test was used to match the mean difference among various groups in Prism 9.0 (GraphPad Software, San Diego, CA, USA). *p* values < 0.05 (two-sided) were recommended as statistically significant.

## 3. Results

### 3.1. RNF7 Is Upregulated in PC Cells and Predicted a Poor Prognosis

**Figure 1:**
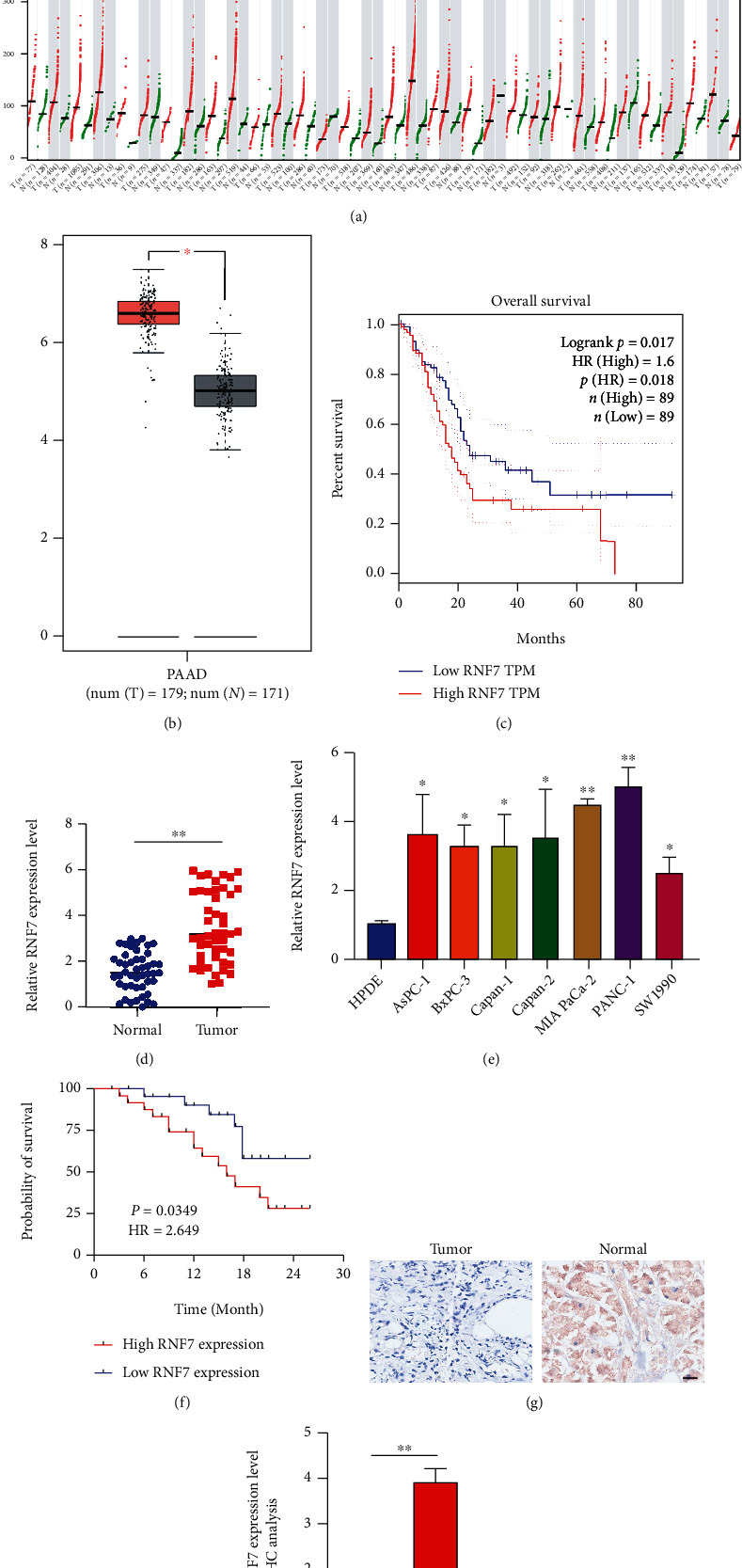
RNF7 is upregulated in PC tissues and demonstrated poor prognosis. (a) The level of RNF7 expression in different cancer tissues compared to normal tissues. (b) Box plots of SOX1 expression in PC tissues compared to normal bile duct tissues. Data sourced from GEPIA database. (c) Kaplan–Meier plot of overall survival of PC patients with RNF7 built on the GEPIA database. (d) RNF7 mRNA expression levels in normal pancreatic tissues and pancreatic cancer tissues. (e) RNF7 mRNA expression levels in normal pancreatic cell line and seven different established PC cell lines. (f) Kaplan–Meier analysis of the association of high- or low-RNF7 expression with overall survival in PC. (g) Representative immunohistochemical staining images of RNF7 expression in PC tissues and matched adjacent nontumor tissues (*n* = 47) (scar bar: 50 *μ*m). (h) Relative RNF7-expression levels in immunohistochemical staining analysis. Data appeared to be average ± SD, ^∗^*p* < 0.05, and ^∗∗^*p* < 0.01.

After analyzing the GEPIA database, the result identified an increased expression of RNF7 in PC tissue compared with normal tissues and is associated with poor prognosis (*p* < 0.05) (Figures 1(a)–1(c)).

To confirm whether RNF7 is related to oncogenesis of PC, we performed qRT-PCR to exploreRNF7 expression in human PC cells. We unveiled that RNF7 expression was higher in human PC cells compared with HPDE (*p* < 0.05) (Figures 1(d) and 1(e)). Meanwhile, based on Kaplan–Meier survival analysis, patients with higher RNF7 levels showed performance in much more terrible overall survival and disease-free survival (*p* < 0.05) (Figure 1(f)). The RNF7 expression in 47 PC tissues was detected by immunohistochemistry staining. The results showed that RNF7 expression harbored the most obvious increase in PC tissues from patients (*p* < 0.01) (Figures 1(g) and 1(h)).

### 3.2. RNF7 Was Involved in PC Proliferative, Migrative, and Invasive Properties

**Figure 2:**
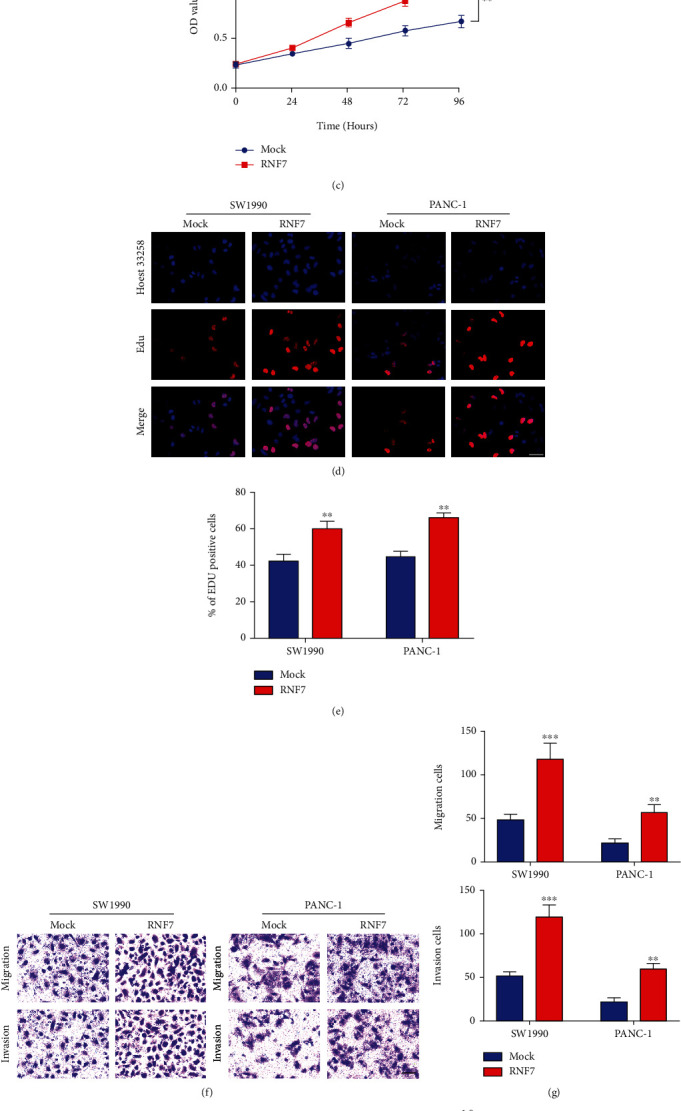
Overexpression of RNF7 promoted the proliferation, invasion, and migration of PC cells. (a) The transfection efficiency of RNF7 was measured by qRT-PCR in PC cells. (b, c) The effect of RNF7 on the proliferation of PC cells was analyzed by CCK-8 assay. (d, e) The EdU assay analyzed the proliferation of PC cells. (f, g) The effect of RNF7 on the invasion of PC cells was analyzed by Transwell assay. (h, i) The effect of RNF7 on the migration of PC cells was analyzed by wound-healing assay (scar bar: 50 *μ*m). ^∗∗^*p* < 0.01; ^∗∗∗^*p* < 0.001.

**Figure 3:**
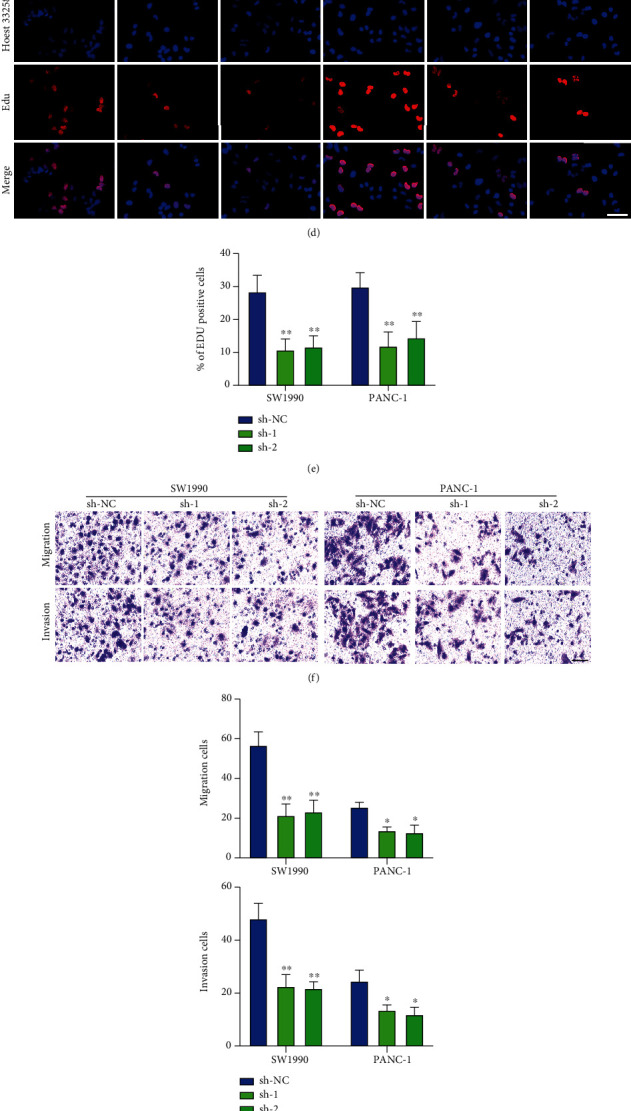
Downregulated RNF7 reduced the proliferation, invasion, and migration of PC cells. (a) Relative expression of RNF7 in PC cells (SW1990 and PANC-1) following transfection of shRNA1, shRNA2, and negative control (sh-NC). (b, c) CCK-8 analysis of the cell proliferative potential after the transfection with RNF7 shRNAs treatment in SW1990 and PANC-1 cell lines. (d, e) The EdU assays analyzed PC proliferative property (SW1990 and PANC-1). (f, g) Transwell analysis of the cell invasive potential after the transfection with RNF7 shRNAs treatment in SW1990 and PANC-1 cell lines. (h, i) Wound-healing analysis of the cell migrative potential after the transfection with RNF7 shRNAs treatment in SW1990 and PANC-1 cell lines (scar bar: 50 *μ*m). ^5^; ^∗∗^*p* < 0.01.

According to RT-qPCR experiment, the result verified the transfection efficiency of RNF7 overexpression (*p* < 0.001) (Figure 2(a)). The results of CCK-8 assay showed that overexpressing RNF7 significantly facilitated PC cell growth (*p* < 0.01) (Figures 2(b) and 2(c)), which supported that RNF7 might have a vital effect in PC development. The similar result was observed for EdU assay (*p* < 0.01) (Figures 2(d) and 2(e)). We performed Transwell and wound-healing assays to explore the invasion and migration of RNF7 in PC cells, and we found that overexpressed RNF7 remarkably promoted their migrative and invasive capacity (*p* < 0.01) (Figures 2(f) and 2(i)). Meanwhile, we conducted a knockdown experiment with SW1990 and PANC-1cells. Specific shRNAs (shRNA1 and shRNA2) were applied for decreasing RNF7 expression within SW1990 and PANC-1 cell lines, and the expression of RNF7 was monitored by qRT-PCR (*p* < 0.01) (Figure 3(a)). Then, we conducted CCK-8 and EdU experiments. The results indicated that RNF7 knockdown dramatically attenuated their proliferation characteristics (*p* < 0.01) (Figures 3(b)–3(e)). In addition, together with cell migration and invasion, Transwell/wound-healing assays, the results of analysis demonstrated that the wound edge of the sh-NC cells was significantly closer than that of the RNF7 low-expressed cells, further revealed that knockdown of RNF7 inhibited PC cell migration and invasion (*p* < 0.05) (Figures 3(f)–3(i)). Above all, RNF7 affected PC oncogenesis in vitro.

### 3.3. RNF7 Is Involved in Cell Proliferation by Activating PI3K/Akt Signaling Pathway

**Figure 4:**
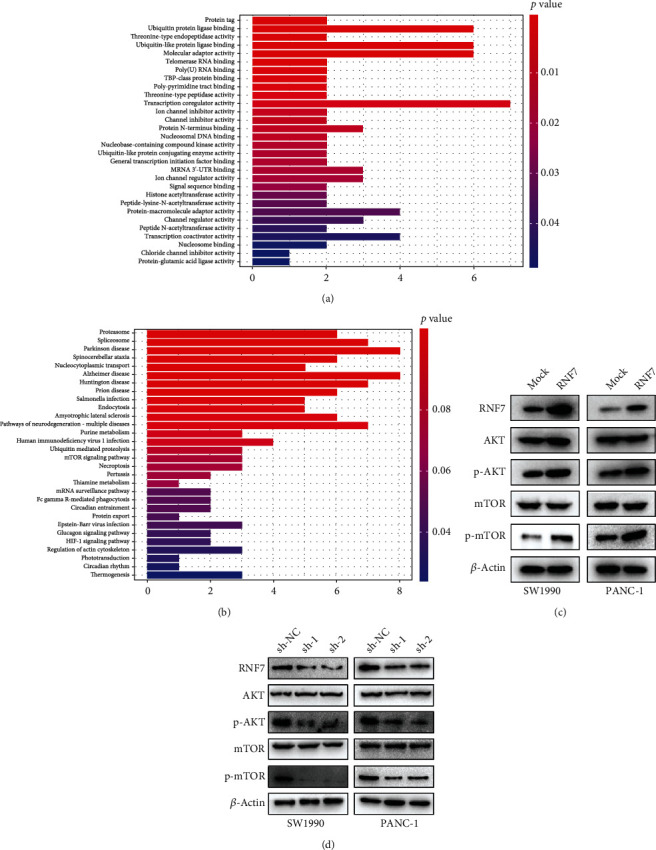
RNF7 promotes cell growth by activating the PI3K/Akt signaling pathway in PC cells. (a, b) GO enrichment analysis of similar genes in the BP, MF, and CC categories and KEGG signaling pathways (KEGG analysis) enriched with the similar genes. (c) Western blot analysis of the activity of PI3K/Akt signaling in PC cellular lineage (SW1990 and PANC-1) when RNF7 was upregulated. (d) Western blot analysis of the activity of PI3K/Akt signaling in PC cellular lineage (SW1990 and PANC-1) when RNF7 was downregulated.

According to the functional enrichment results, similar genes of RNF7 (Supplementary File 1) in GEPIA (Gene Expression Profiling Interactive Analysis) (cancer-pku.cn) database were enriched in the GO enrichment analysis (Figure 4(a)). The KEGG analysis suggested that those genes could be enriched in the mTOR pathway (Figure 4(b)). To explore whether PI3K/Akt/mTOR pathway participated in the proliferation, migration, and invasion of PC cells, we conducted western blotting to identify the expression of PI3K/AKT-related protein. We found that although the total AKT and mTOR expression were not changed, overexpression of RNF7 increased the expression of both active phospho-Akt and phospho-mTOR (Figure 4(c)) and the levels of phospho-Akt, and phospho-mTOR decreased following downregulated RNF7 expression in PC cells (Figure 4(d)). Above all, RNF7 exerts migration and invasion effects by activating PI3K/Akt signaling pathway in PC cells.

### 3.4. RNF7 Accelerates the Progression of PC through PI3K/AKT Signal Pathway

**Figure 5:**
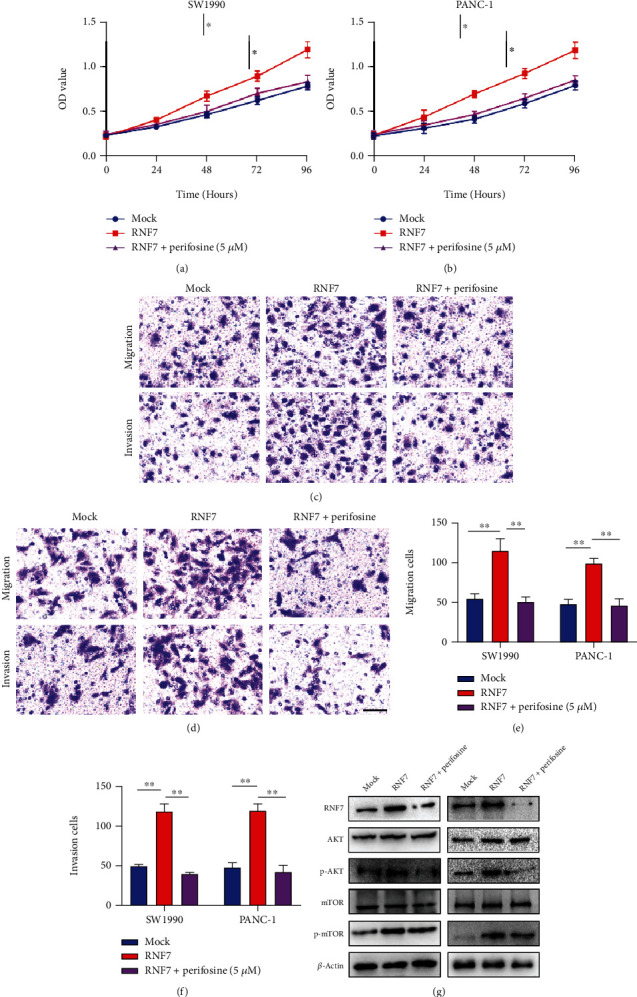
The rescue experiment of the effect of RNF7 on proliferation, migration, and invasion of PC cells. (a, b) Cell proliferation assay of SW1990 and PANC-1 cells. Overexpression of RNF7 promoted the proliferation ability and the inhibitor of perifosine inhibited this effect. (c–f) Transwell assay of SW1990 and PANC-1 cells. Overexpression of RNF7 promoted the invasion ability and the inhibitor of perifosine inhibited this effect (scar bar: 50 *μ*m). (g) Protein expression levels and quantitative data of active phospho-Akt and phospho-mTOR in SW1990 and PANC-1 cells were shown. Overexpression of RNF7 activated the PI3K/AKT pathway, and the signaling inhibitor perifosine counteracted the activation.

We conducted the rescue experiment to further confirm the essential role of PI3K/AKT signal pathway in RNF7-mediated PC progression with the help of pathway inhibitor perifosine. After treatment with RNF7 in the presence or absence of inhibitor perifosine, the CCK-8 experiment revealed that perifosine significantly attenuated RNF7-induced cell proliferation compared to that in non-PI3K-inhibitor-treated cells (*p* < 0.05) (Figures 5(a) and 5(b)). The Transwell and wound-healing assay showed that overexpression of RNF7 enhanced the corresponding with that in the control group; in contrast, the opposite results exhibited in the group of PC cells treated with the PI3K/AKT signaling pathway inhibitor perifosine (5 *μ*M) (*p* < 0.01) (Figures 5(c)–5(f)). The signaling pathway detection also demonstrated phospho-Akt and phospho-mTOR upregulation, with such influences being rescued perifosine inhibitor treatment (Figure 5(g)). Our data confirmed that RNF7 might contribute to accelerating PC cell tumorigenic progression by activating PI3K/Akt signaling pathway.

### 3.5. RNF7 Promoted the Tumorigenic Capacity of PC In Vivo

**Figure 6:**
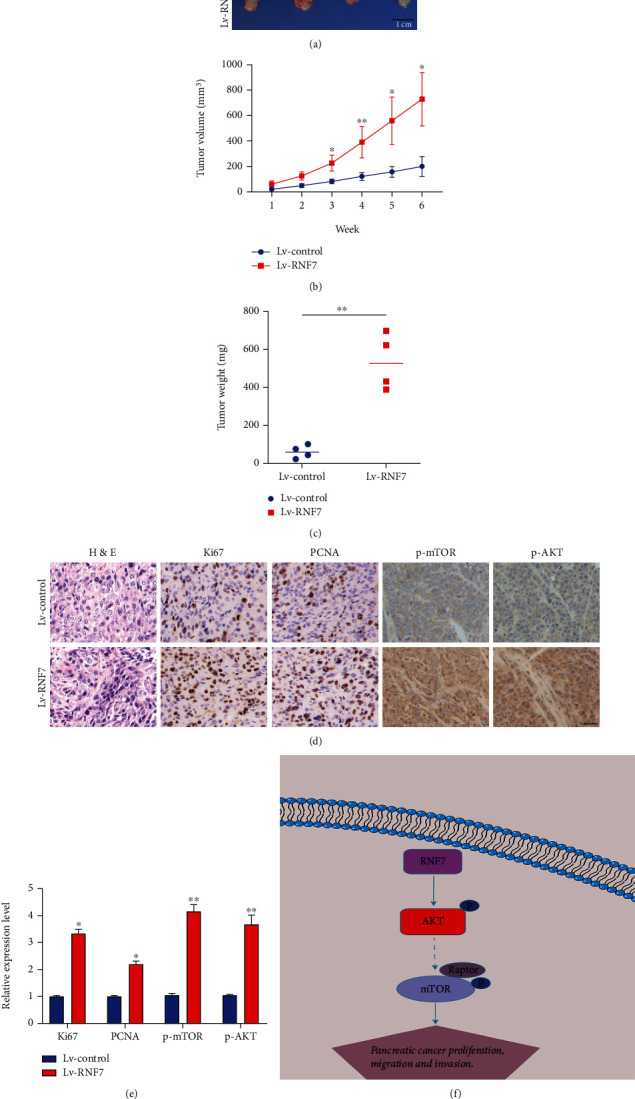
RNF7overexpression accelerated tumor growth in vivo. (a) Xenograft tumors were removed from Lv-RNF7 or Lv-NC groups. (b, c) Tumor volume and weight in Lv-RNF7 or Lv-NC groups were measured and calculated. (d) Immunohistochemical staining for Ki-67, PCNA, phospho-Akt, and phospho-mTOR in xenografts (scar bar: 50 *μ*m). (e) qRT-PCR was used to detect the expression levels of central members of PI3K/Akt signaling in each group. (f) A schematic illustration of the mechanism by which RNF7 promotes progression of PC. ^∗^*p* < 0.05; ^∗∗^*p* < 0.01.

To confirm whether RNF7 exerts promoting effect in tumor development in vivo, we established a subcutaneous tumorigenesis model by injecting pancreatic cancer cells with stable overexpression of RNF7 or control cells into mice. As showed in Figure 6(a), intratumorally injection of cells transfection of RNF7 overexpression lentivirus markedly increased tumor volume and weight compared with control mice. Moreover, the volume and weight of mice grew faster in the RNF7-overexpressed group compared with the control group (*p* < 0.01) (Figures 6(b) and 6(c)). Furthermore, histological analysis revealed that Ki-67, PCNA, phospho-Akt, and phospho-mTOR staining increased in the tumors from RNF7-overexpressed group compared with control tumors (Figure 6(d)). Cases with RNF7 high-expression also possessed a positive correlation with the high level of Ki-67, PCNA, phospho-Akt, and phospho-mTOR protein expression (*p* < 0.05) (Figure 6(e)). These results are in lined with the effects of RNF7 in vitro.

## 4. Discussion

Pancreatic cancer is characterized by high mortality and obvious aggressiveness for the past 30 years [15, 17]. At present, the major treatment for PC is surgical resection, while postoperative patients and patients with advanced disease, despite treated by subsequently adjuvant therapies, are difficult to avoid high-recurrence rates and poor treatment effectiveness [18]. Therefore, advancing our understanding of the underlying cancer-promoting events of PC may contribute to find its effective therapeutic targets. RNF7, consisted of 113 amino acids, participates in posttranslational modification. It is well known that the deubiquitination of MALT1 and NEMO stimulated by RNF7 expression exerts a critical role in activation of NF-kB by RNF proteins to regulate CARM [19–22]. It has been reported that RNF7 acts as an oncogenic cooperator to promote numerous cancers [23], and the high-expression level of RNF7 is associated with poor prognosis of cancer patients [24, 25]. In addition, it was reported that RNF7 exhibits an oncogenic role in the development of prostate cancer interacting with PTEN-loss via activating the PI3K/Akt/mTOR signaling pathway [26]. In a credible Kras^G120D^-lung tumorigenesis model, RNF7 is a fundamental element for Kras-induced lung tumorigenesis [27]. The condition of serum-hunger stress also significantly influence the effect of RNF7 on promotion of S-phase entry and cell growth [28]. However, in reviewing the literature, few data were found on its role in the PC. Therefore, we conducted this study to investigate the effect of RNF7 on PC. To understand the role of RNF7 in PC, we first examined the expression of RNF7 in PC clinical specimens and the data on the public GEPIA database. The results showed upregulation of RNF7 in PC tissues. Through exploring the prognosis of PC patients, we found that high level of RNF7 was associated with poor overall survival (*p* < 0.05), which indicate that RNF7 may be associated with tumorigenesis of PC. In this context, we applied RNF7-upregulated and RNF7-silenced systems to determine the functions and mechanism of RNF7 in PC, and we found that ectopic expression of RNF7 promoted the tumorigenesis of PC cells, while silencing of RNF7 attenuated these effects in vitro and in vivo. Our findings indicated RNF7 might be an oncogenic factor in PC.

The interference of signaling pathways in human cancer provides a new field of cancer treatment [29–31]. The potential molecular mechanism of the association of RNF7 with PC was also investigated in this research. PI3K/AKT signaling pathway, which is frequently deregulated in human cancers, has been proven to be central in mediating a variety of cellular processes, including cell proliferation, apoptosis, and cell migration [32–34]. Prior studies have noted the importance of PI3K/AKT signaling pathway on promoting the development of renal cancer [35, 36]. It has been shown that PI3K/Akt signaling pathway was well correlated with the regulation of lncRNA NEAT1 in cervical carcinoma proliferation and invasion [37]. Guo et al. suggested that ROR2/PI3K/Akt regulatory network might contribute to promote the progression of breast cancer [38]. Analysis of mice xenograft models by Xun et al. also reported that lncRNA HOTAIR modulated gastric cancer progression through PI3k/ATK signaling pathway [39]. The mechanistic target of rapamycin (mTOR) is a dual specificity protein kinase composed by several distinct protein complexes with the capability of promoting cell growth and cell-cycle progression [40]. Among the mTOR complex, mTOR complex 1 (mTORC1) becomes one of dominant downstream targets in PI3K/ATK pathway and promotes protein synthesis by phosphorylates key regulators of mRNA translation and ribosome synthesis to complete protein synthesis [41]. PI3k/ATK/mTOR signaling pathway was found to be relevant across the metabolism proliferation, invasion, and metastasis of tumor cells [42]. In breast cancer, almost 60% of tumors exhibit alternations in this signaling route, and changes in the signaling pathway are common in cancer [43]. Moreover, mounting researches on this pathway are beneficial for an effective drug development and cancer therapy [44]. Thus, ATK expression may reflect the proliferative ability of the tumor, and its overactivation plays an essential role in cancer development and progression. Interestingly, the result of KEGG found that RNF7 is associated with PI3k/ATK signaling pathway in PC. Then, we explored the relationship between RNF7 and PI3k/ATK pathway. We observed that overexpression of RNF7 activated the PI3k/ATK pathway, leading to increased proliferation of PC cells. However, downregulated of RNF7 expression reduced the activation of the pathway, resulting in inhibiting PC oncogenesis. Furthermore, pretreatment of cells with a PI3K inhibitor had been instrumental in positively preventing cell proliferation, migration, and invasion compared to that in control group. These data indicated that RNF7 promoted PC through its effect on the activation of PI3k/ATK signaling pathway.

## 5. Conclusions

Our data demonstrated that RNF7 expression is overexpressed in PC tissues and accelerates the malignant progression of PC in vitro and in vivo. Furthermore, RNF7 promotes the tumorigenesis and development of PC via regulating PI3K/Akt signaling pathway. The present study was designed to determine that RNF7 may be the potential therapeutic target of PC.

## Data Availability

The datasets used during the current study are available from the corresponding author on reasonable request.

## Abbreviations

RNF7: RING finger protein-7
SAG: Sensitive to apoptosis gene
ROC2: Regulator of cullins 2
Rbx2: RING-box 2
PC: Pancreatic cancer
AKT: Protein kinase B
p-AKT: Phosphorylated protein kinase
mTOR: Mammalian target of rapamycin
p-mTOR: Phosphorylated mammalian target of rapamycin
PI3K: Phosphatidylinositol 3-kinase
CCK-8: Cell Counting Kit-8
EdU: 5-Ethynyl-20-deoxyuridine
IHC: Immunohistochemical streptavidin-peroxidase staining
HE: Hematoxylin and eosin
PBS: Phosphate buffered saline
qRT-PCR: Quantitative real-time polymerase chain reaction
GEPIA: Gene expression profiling interactive analysis
GO: Gene Ontology
KEGG: Kyoto Encyclopedia of Genes and Genomes.

## Supplementary Materials

Supplementary Materials Supplementary File 1: similar genes of RNF7 in GEPIA database.
